# Supplementary material for: Stable Bicyclic Functionalized Nitroxides: The Synthesis of Derivatives of Aza-nortropinone–5-Methyl-3-oxo-6,8-diazabicyclo[3.2.1]-6-octene 8-oxyls
Source: Molecules. 2021 May 20;26(10):3050. doi: 10.3390/molecules26103050 (PMC8161028; doi:10.3390/molecules26103050)
Supplement: Supplementary file 1 [file molecules-26-03050-s001.zip › molecules-1210799-supplementary.pdf]

molecules

MDPI

SUPPLEMENTARY MATERIALS

for the article

“Stable bicyclic functionalized nitroxides: the synthesis of derivatives of aza-nortropinone-5-methyl-3-oxo-6,8-diazabicyclo[3.2.1]-6-octene 8-oxyls”

L.N. Grigor’eva, A.Ya. Tikhonov, K.A. Lomanovich and D.G. Mazhukin

Content

NMR spectra of bicycles **5a,c,d** and pyridone **3d** S2

IR spectra for bicycles **5a,c,d** and nitroxides **c** S9

HRMS spectra of bicyclic nitroxides **6a,c,d** S12

Selected ESR *hfc* constants for radical **6c** S16

^{1}H and ^{13}C NMR spectra of 8-hydroxy-6,8-diazabicyclo[3.2.1]oct-6-en-3-ones **5**

Compound **5a**

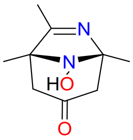

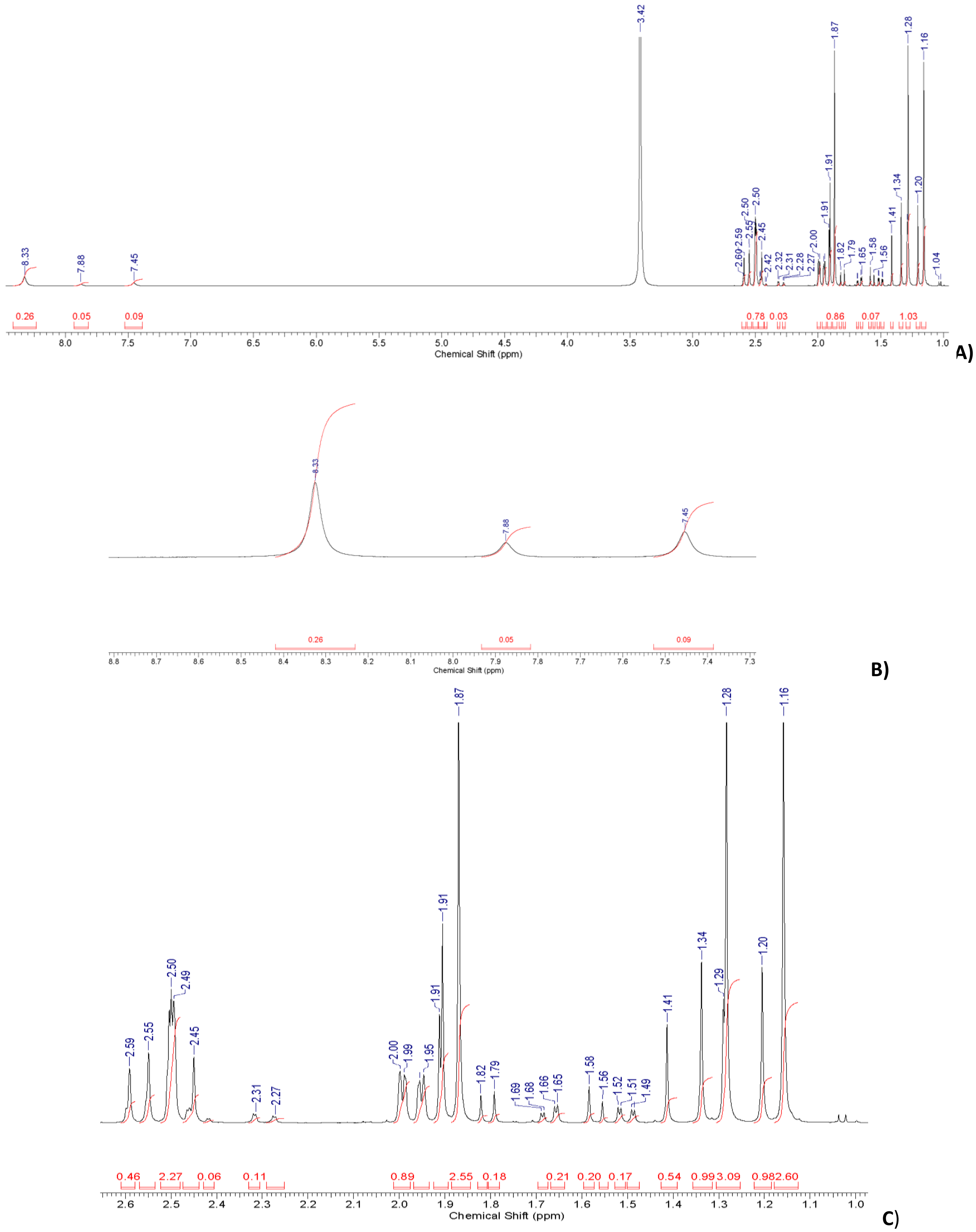

Fig. S1. ^{1}H NMR spectrum of **5a** in DMSO-d_6. A) Whole spectrum; B) Downfield region; C) Upfield region.

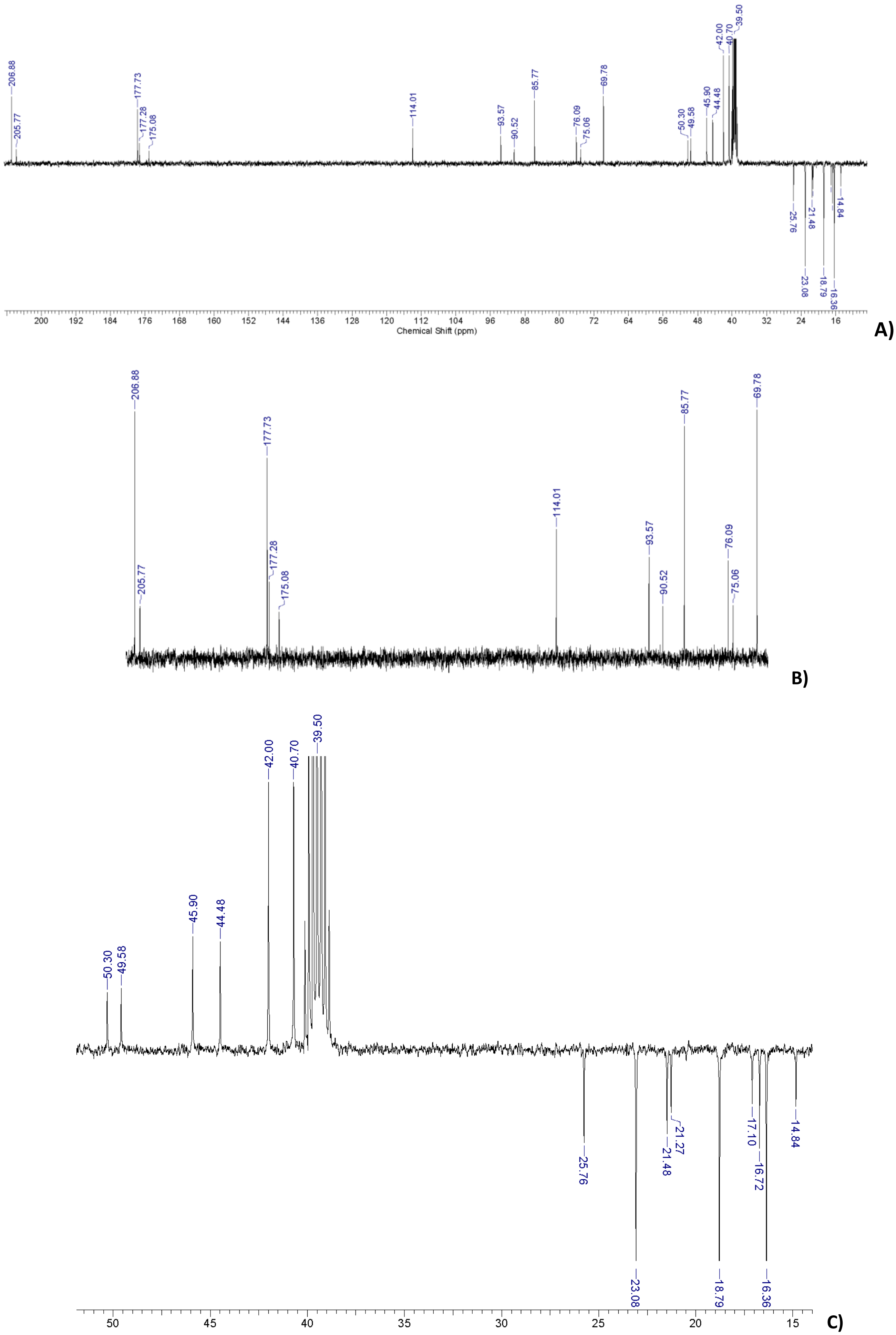

Fig. S2. ^{13}C NMR spectrum of **5a** in DMSO-d_6. A) Whole spectrum; B) Downfield region; C) Upfield region.

Compound **5c**

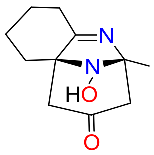

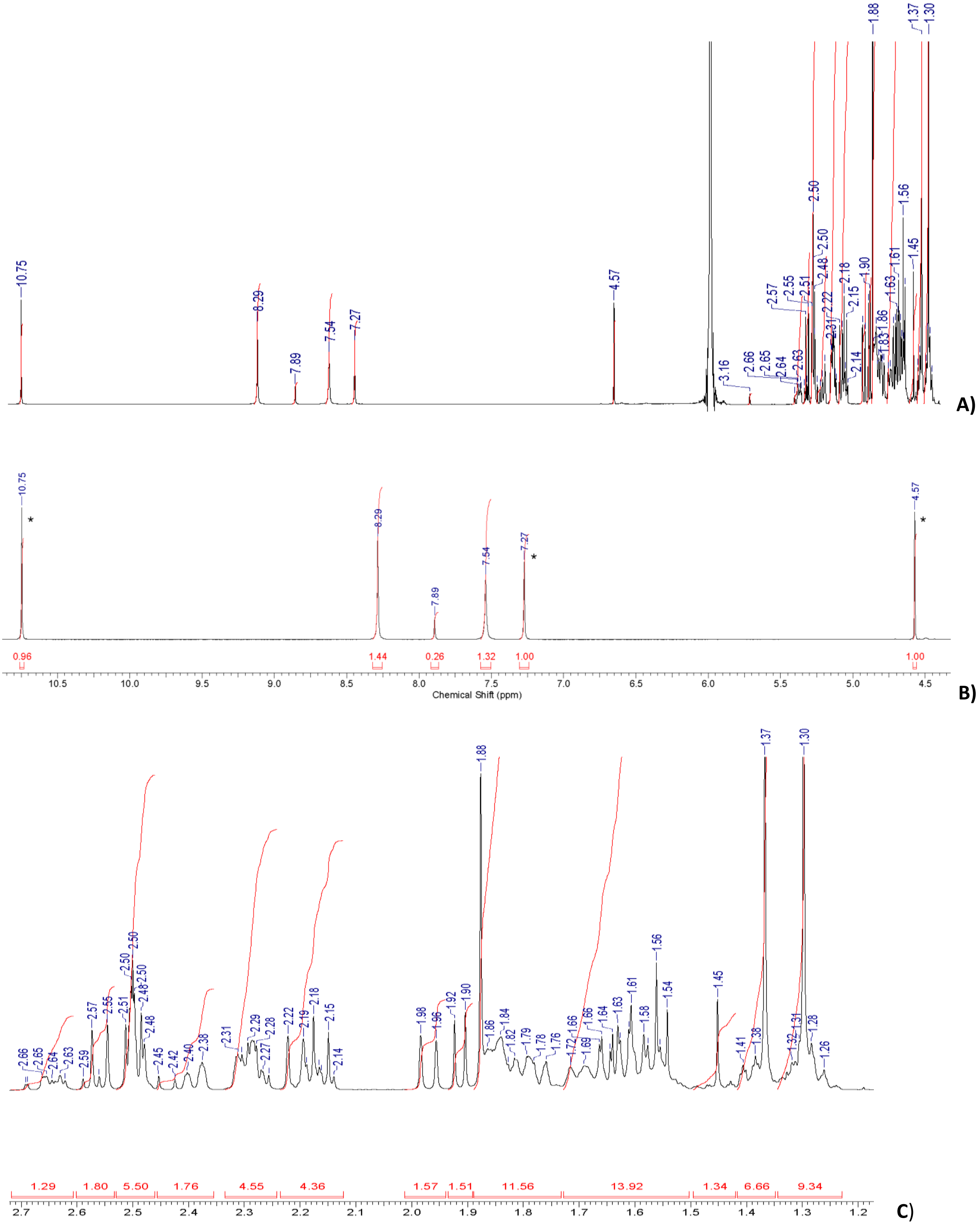

Fig. S3. ^{1}H NMR spectrum of **5c** in DMSO-d_6. A) Whole spectrum; B) Downfield region (* - denotes signals of pyridone **3c** impurity); C) Upfield region.

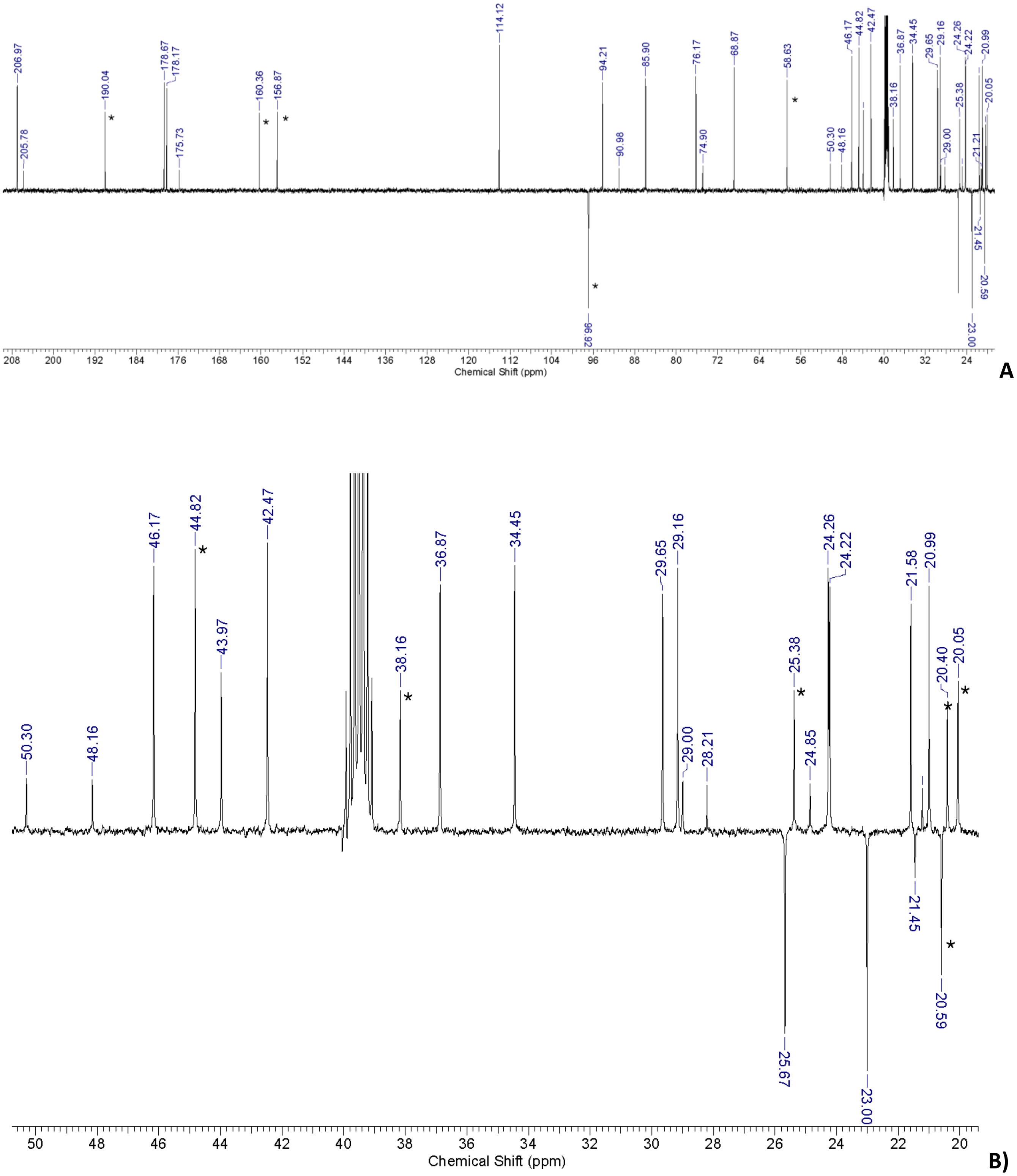

Fig. S4. ^{13}C NMR spectrum of **5c** in DMSO-d_6. A) Whole spectrum; B) Upfield region.
(* - denotes signals of pyridone **3c** impurity)

Compound **5d**

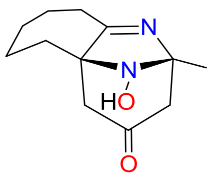

A)

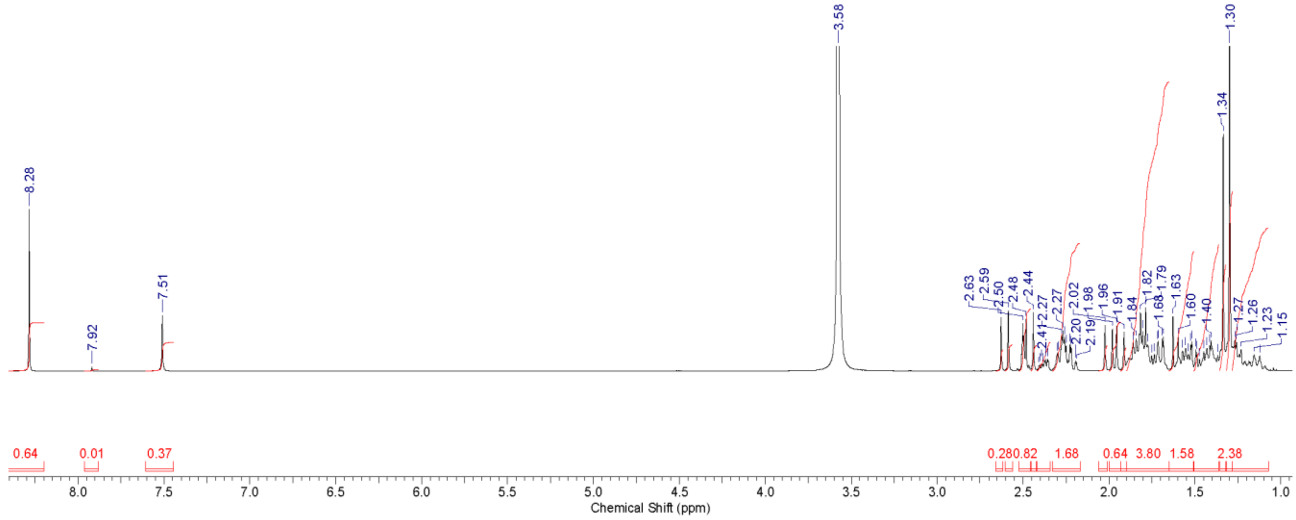

B)

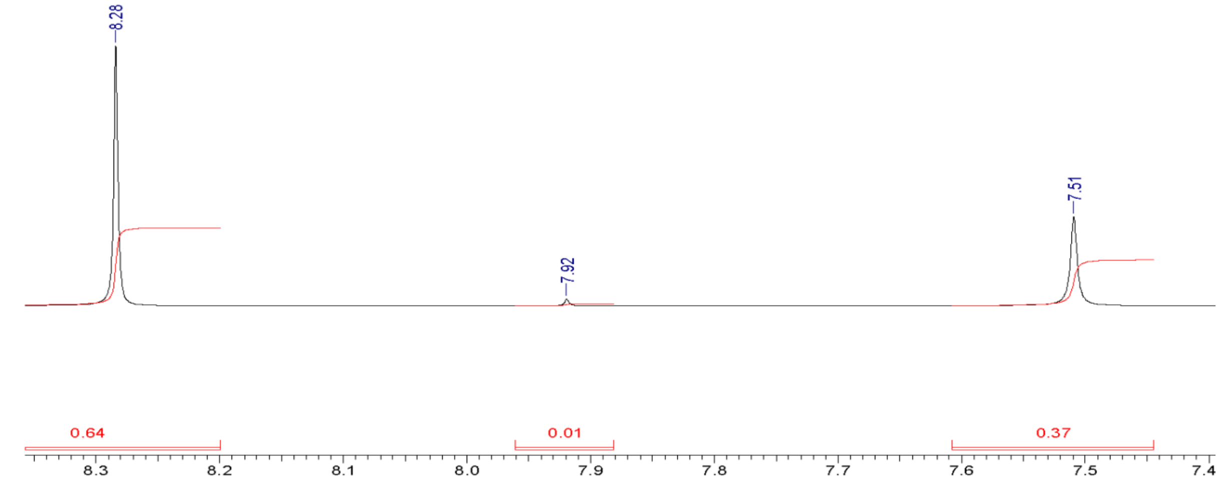

B)

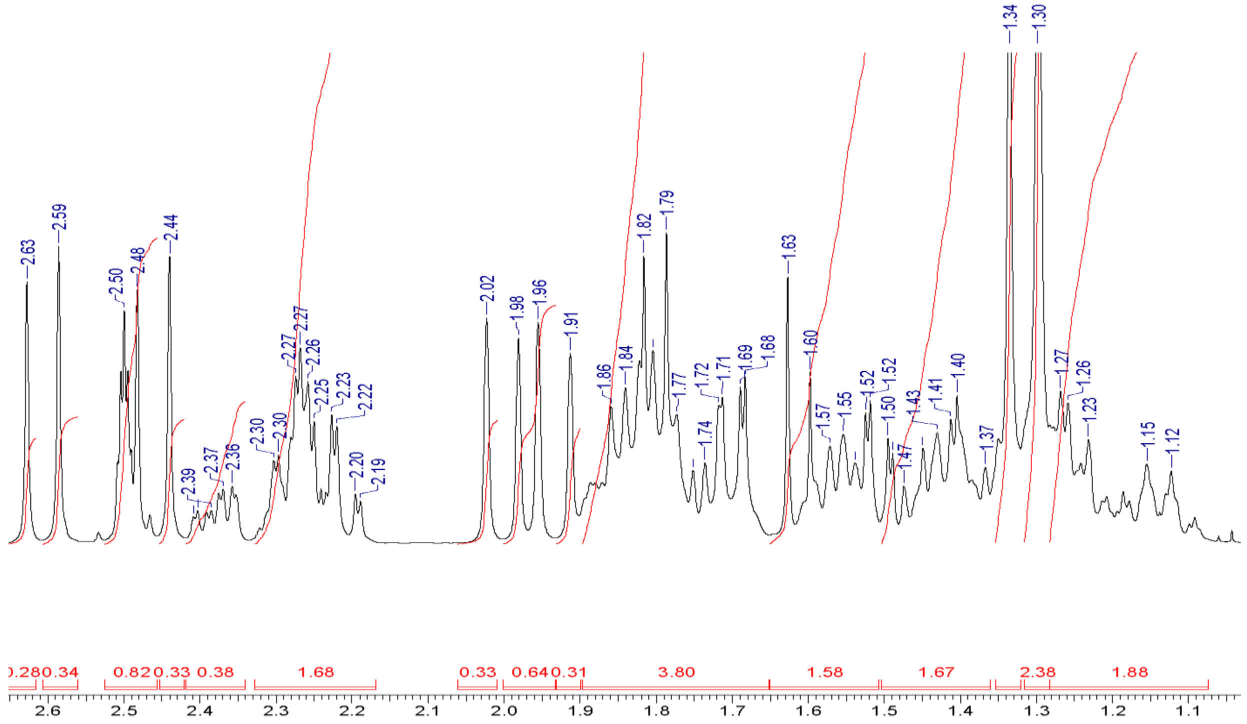

C)

Fig. S5. ^{1}H NMR spectrum of **5d** in DMSO-d_6. A) Whole spectrum; B) Downfield region; C) Upfield region.

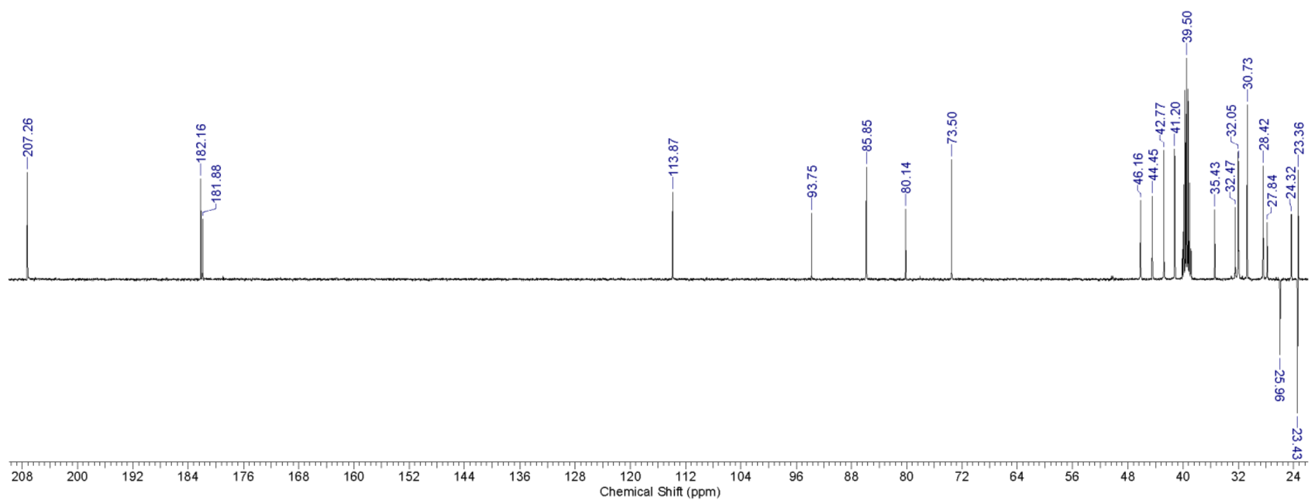

A)

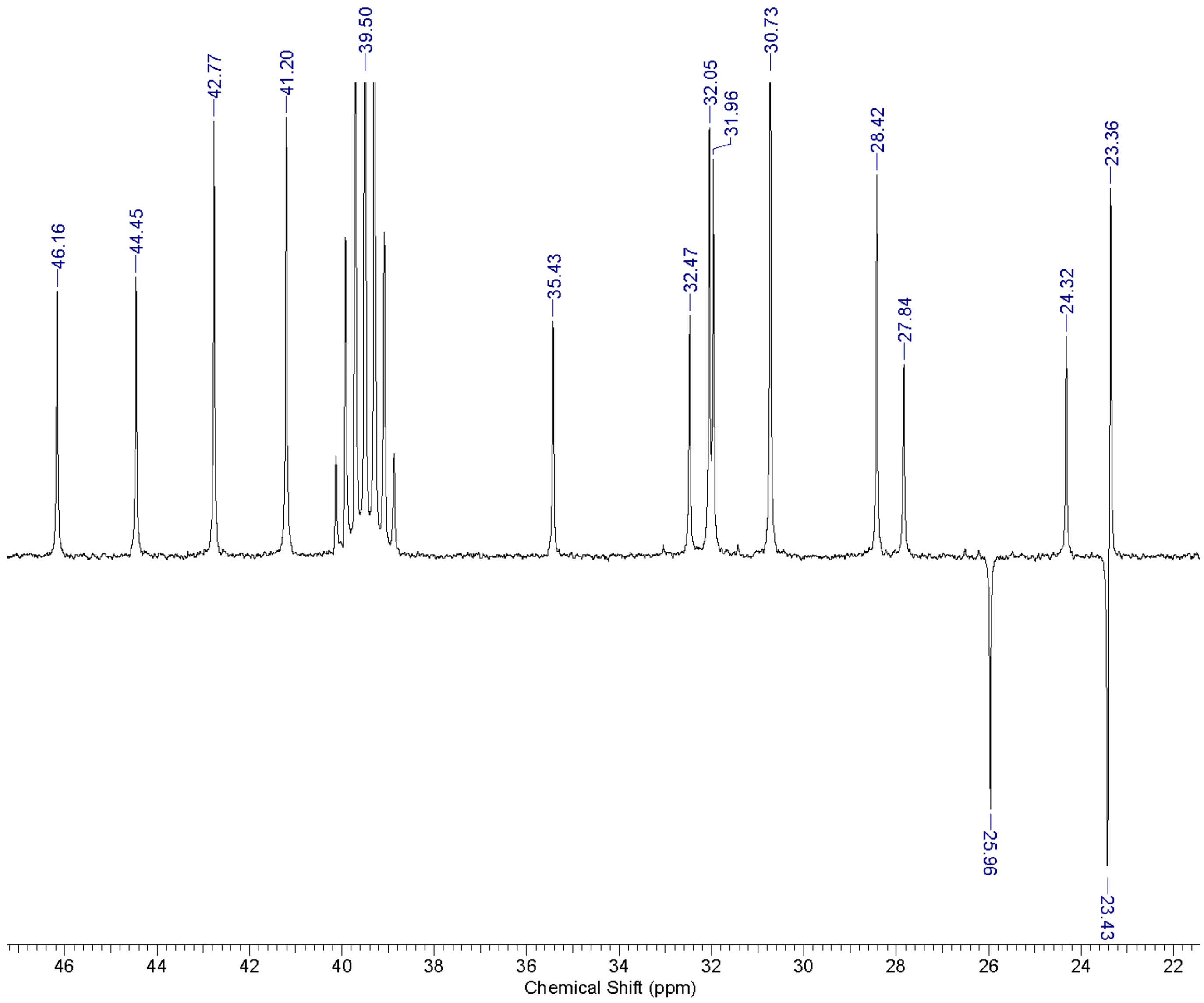

Fig. S6 ^{13}C NMR spectrum of **5d** in DMSO-d_6. A) Whole spectrum; B) Upfield region.

^{13}C NMR spectra of 7-(Hydroxyimino)-2-methyl-1-azaspiro[5.6]dodec-2-en-4-one (**3d**)

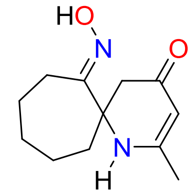

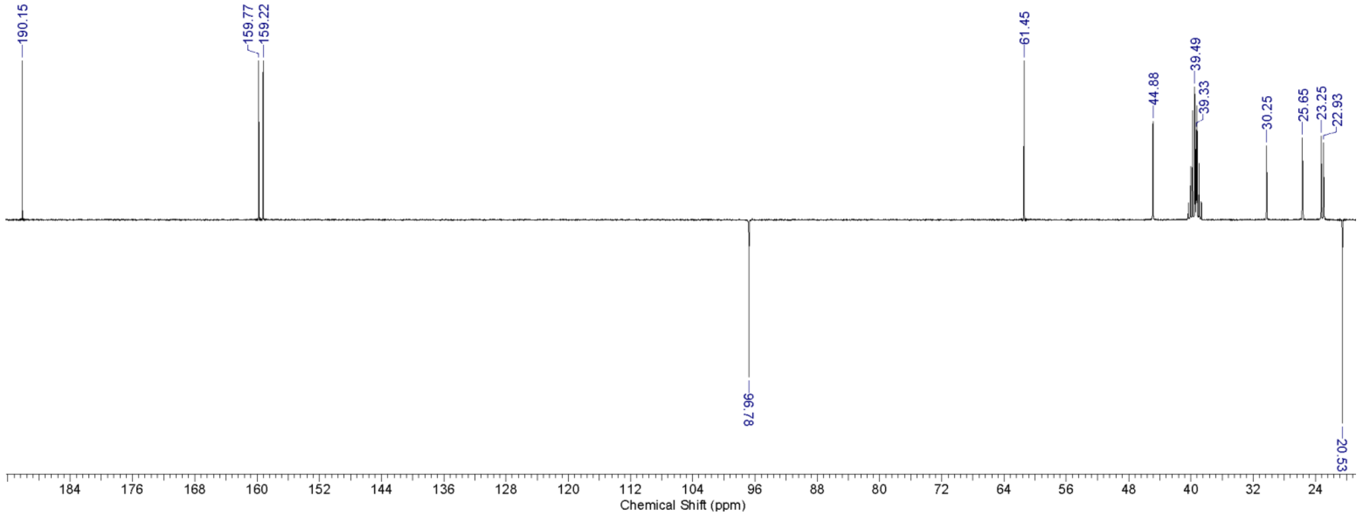

Fig. S7 ^{13}C NMR spectrum of pyridone **3d** in DMSO-d_6.

IR-Spectra of bicycles **5** and **6**

Compound **5a**

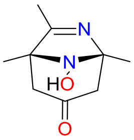

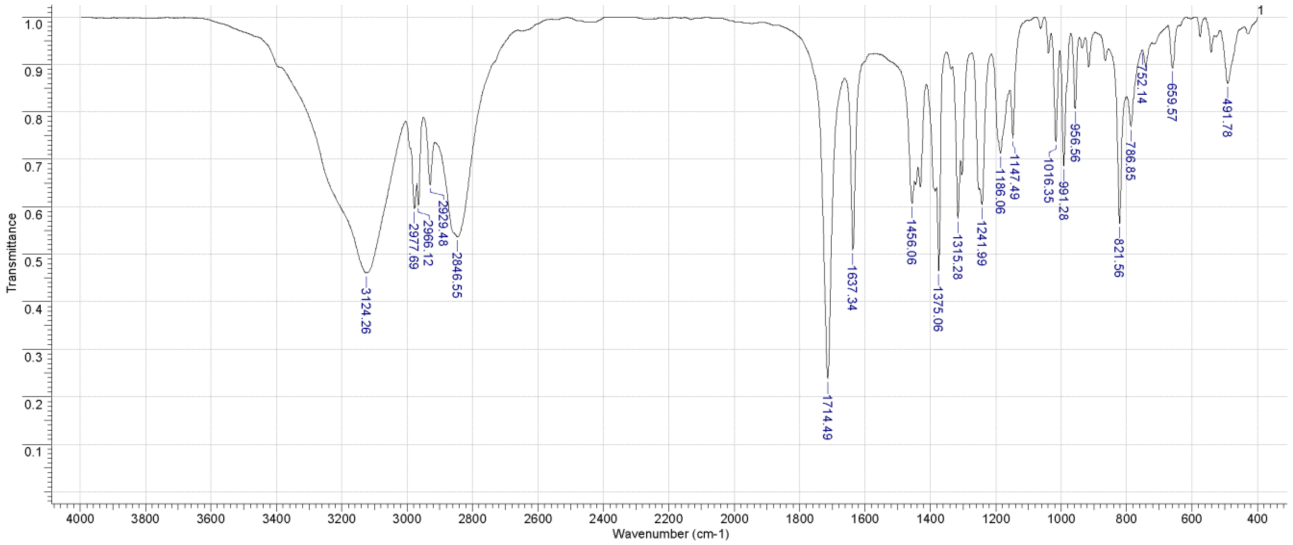

Fig. S8. IR spectrum of **5a** in KBr.

Compound **6a**

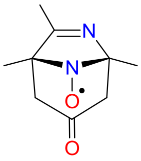

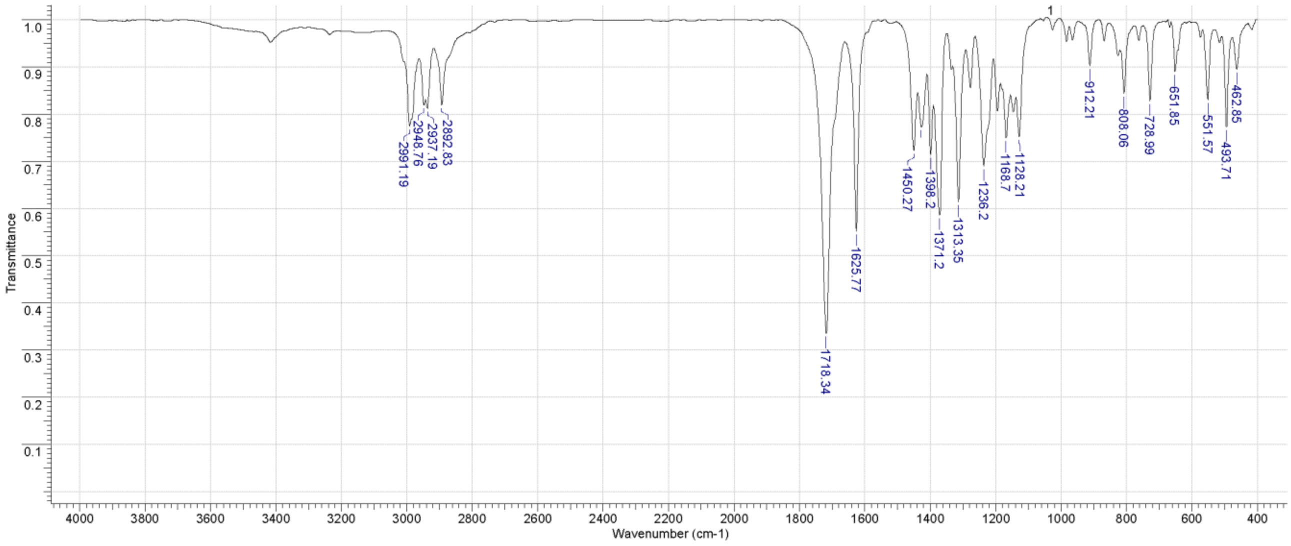

Fig. S9. IR spectrum of **6a** in KBr.

Compound **5c**

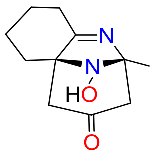

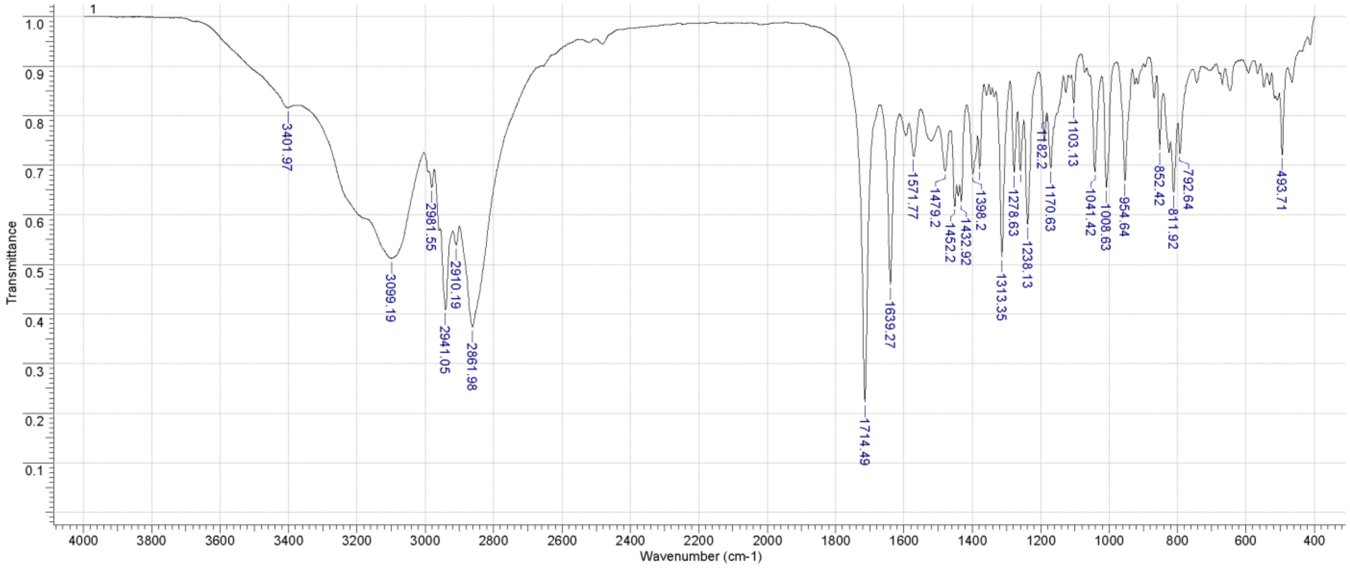

Fig. S10. IR spectrum of **5c** in KBr.

Compound **6c**

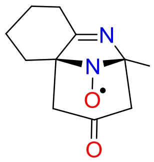

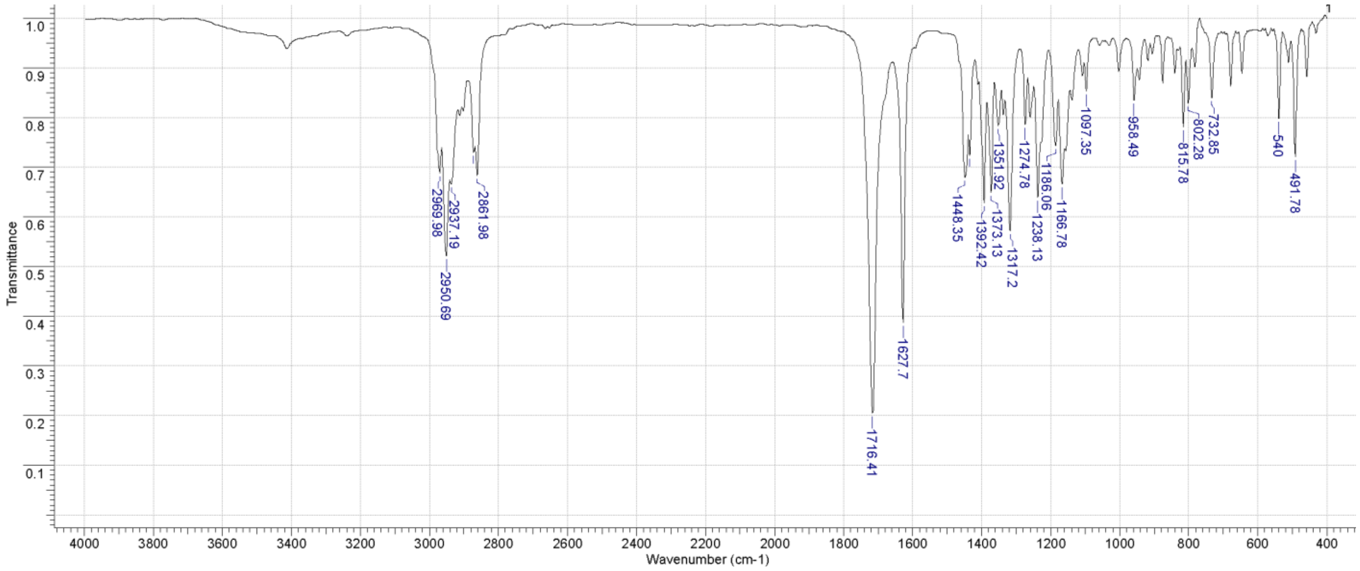

Fig. S11. IR spectrum of **6c** in KBr.

Compound **5d**

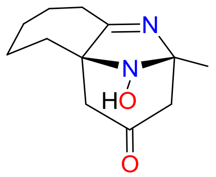

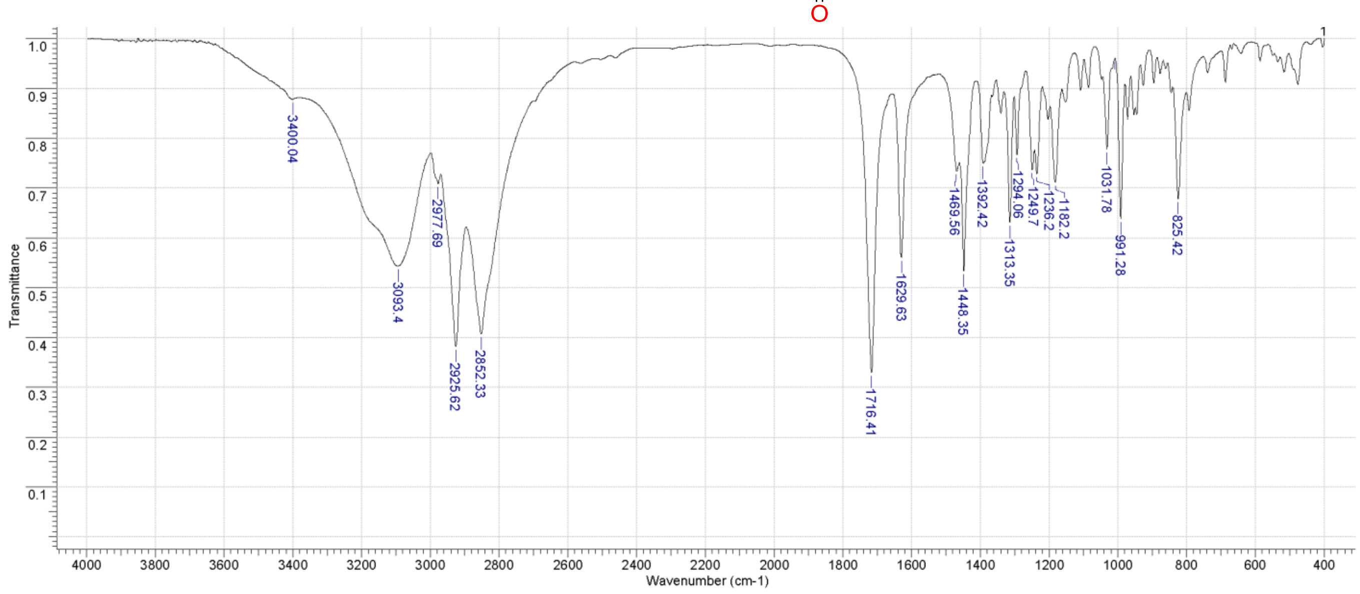

Fig. S12. IR spectrum of **5d** in KBr.

Compound **6d**

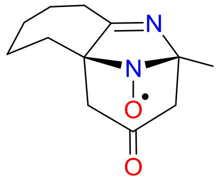

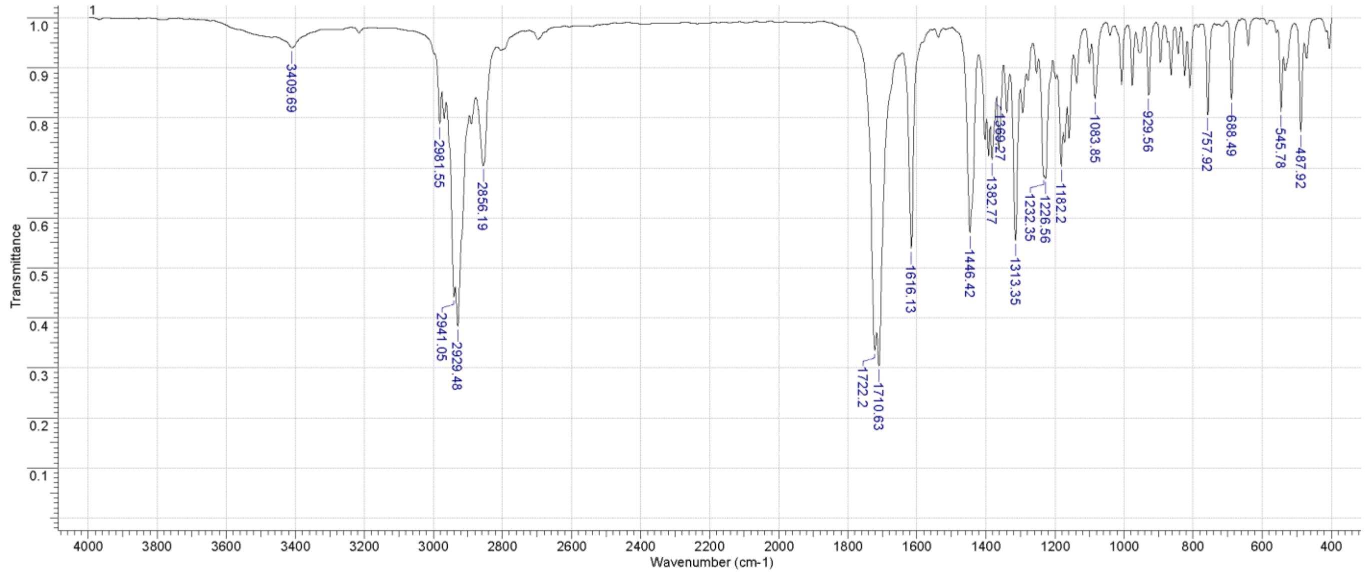

Fig. S13. IR spectrum of **6d** in KBr.

HRMS spectra of bicyclic nitroxyl radicals 6a,c,d

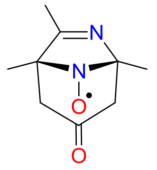

Nitroxide **6a**

T source = 75˚C
(without heating the rod with the sample)

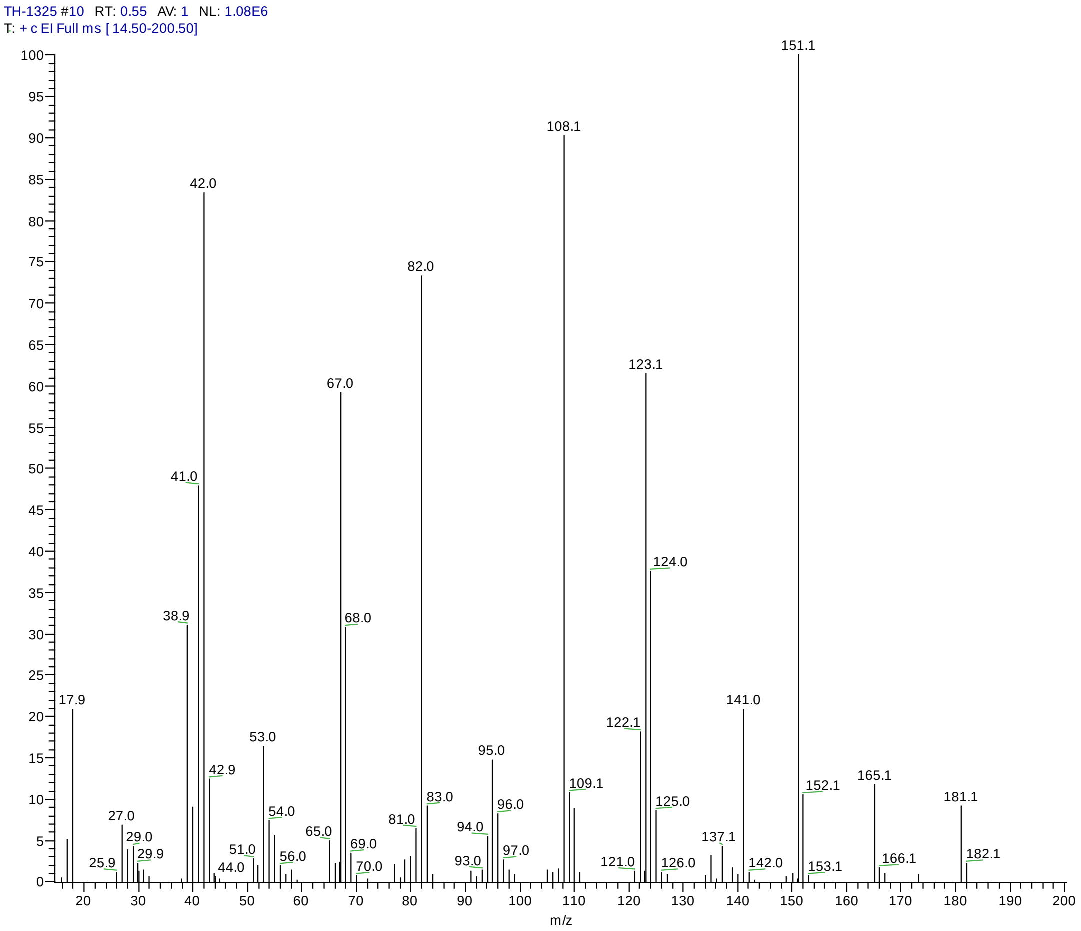

m/z	Intensity	Relative	m/z	Intensity	Relative
17.9	226661.0	20.91	121.0	13272.0	1.22
38.9	337103.0	31.09	122.1	197090.0	18.18
40.0	98201.0	9.06	123.1	666023.0	61.43
41.0	518858.0	47.86	124.0	407660.0	37.60
41.9	88367.0	8.15	125.0	92975.0	8.58
42.0	903825.0	83.37	126.0	12515.0	1.15
42.9	134923.0	12.45	135.1	34040.0	3.14
53.0	177285.0	16.35	137.1	46143.0	4.26
67.0	642023.0	59.22	139.1	18842.0	1.74
68.0	333733.0	30.78	141.0	226729.0	20.91
68.0	109204.0	10.07	142.0	12859.0	1.19
82.0	794345.0	73.27	151.1	1084136.0	100.00
83.0	99576.0	9.18	152.1	113605.0	10.48
95.0	159543.0	14.72	165.1	128047.0	11.81
96.0	89124.0	8.22	166.1	17742.0	1.64
108.1	978645.0	90.27	181.1	99645.0	9.19
109.1	117319.0	10.82	182.1	23656.0	2.18
110.1	96275.0	8.88			

Calculation of elemental compositions

Calculated value m/z= 181.0972 ($C_9H_{13}O_2N_2$)$^{+\cdot}$
Measured value m/z= 181.0970

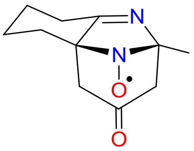

Nitroxide **6c**

T source = 50°C
(without heating the rod with the sample)

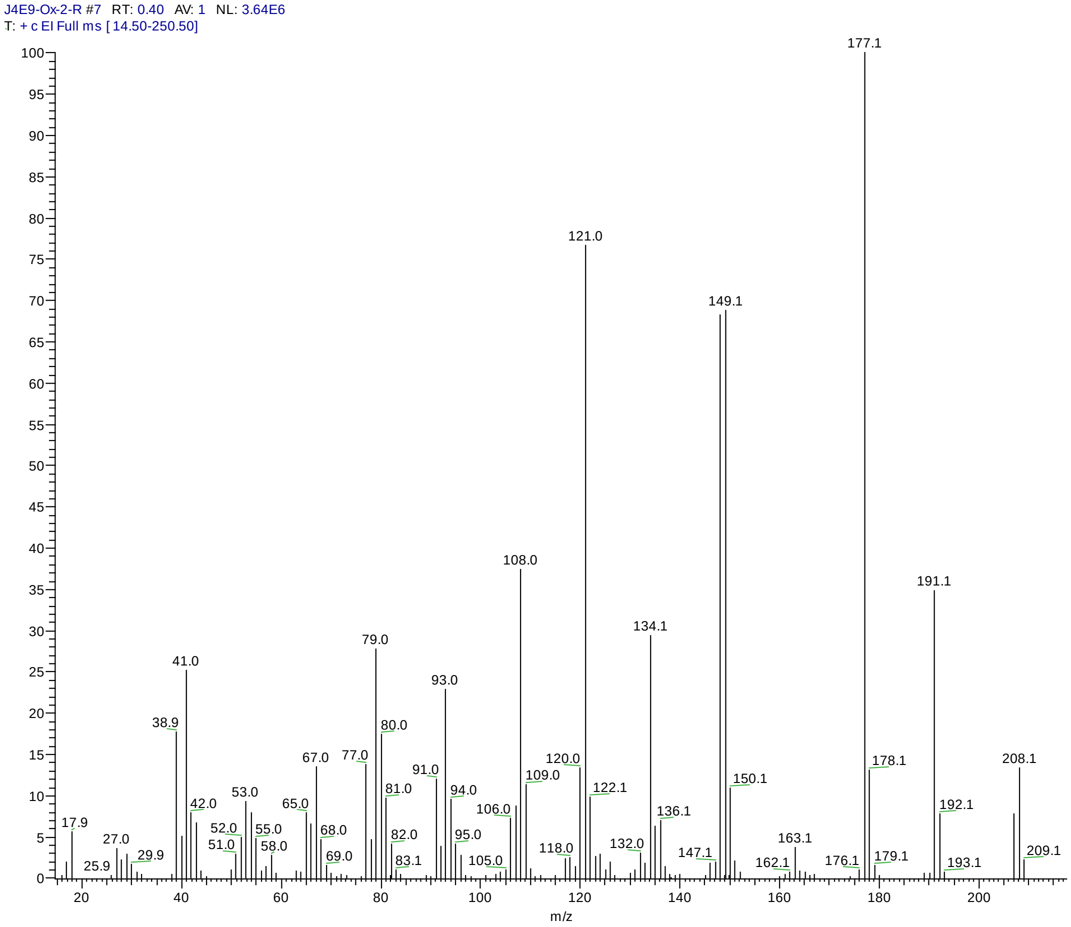

m/z	Intensity	Relative	m/z	Intensity	Relative
17.9	204004.0	5.60	96.0	244823.0	8.22
38.9	645620.0	17.74	106.0	265360.0	7.29
41.0	919796.0	25.27	108.0	1361590.0	37.40
42.0	288701.0	7.93	109.0	413778.0	11.37
53.0	336389.0	9.24	120.0	487440.0	13.39
54.0	287488.0	7.90	121.0	2793440.0	76.74
55.0	175309.0	4.82	134.1	1071439.0	29.43
65.0	288878.0	7.94	135.1	230304.0	6.33
67.0	491167.0	13.49	136.1	254177.0	6.98
68.0	110322.0	30.78	148.1	2486634.0	68.31
68.0	171729.0	4.72	149.1	2505834.0	68.84
77.0	501551.0	13.78	150.1	397211.0	10.91
79.0	1012953.0	27.83	177.1	3640197.0	100.00
80.0	634113.0	17.42	178.1	477677.0	13.12

91.0	438805.0	12.05	191.1	1267368.0	34.82
93.0	833473.0	22.90	192.1	284441.0	7.81
94.1	285419.0	16.48	207.1	283997	7.80
95.1	115977.0	6.70	208.1	486819.0	13.37

Calculation of elemental compositions

Calculated value m/z= 207.1128 $(C_{11}H_{15}O_2N_2)^{+\cdot}$

Measured value m/z= 207.1126

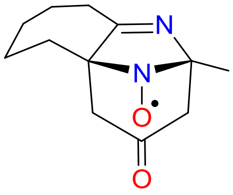

Nitroxide **6d**

T source = 70°C

(without heating the rod with the sample)

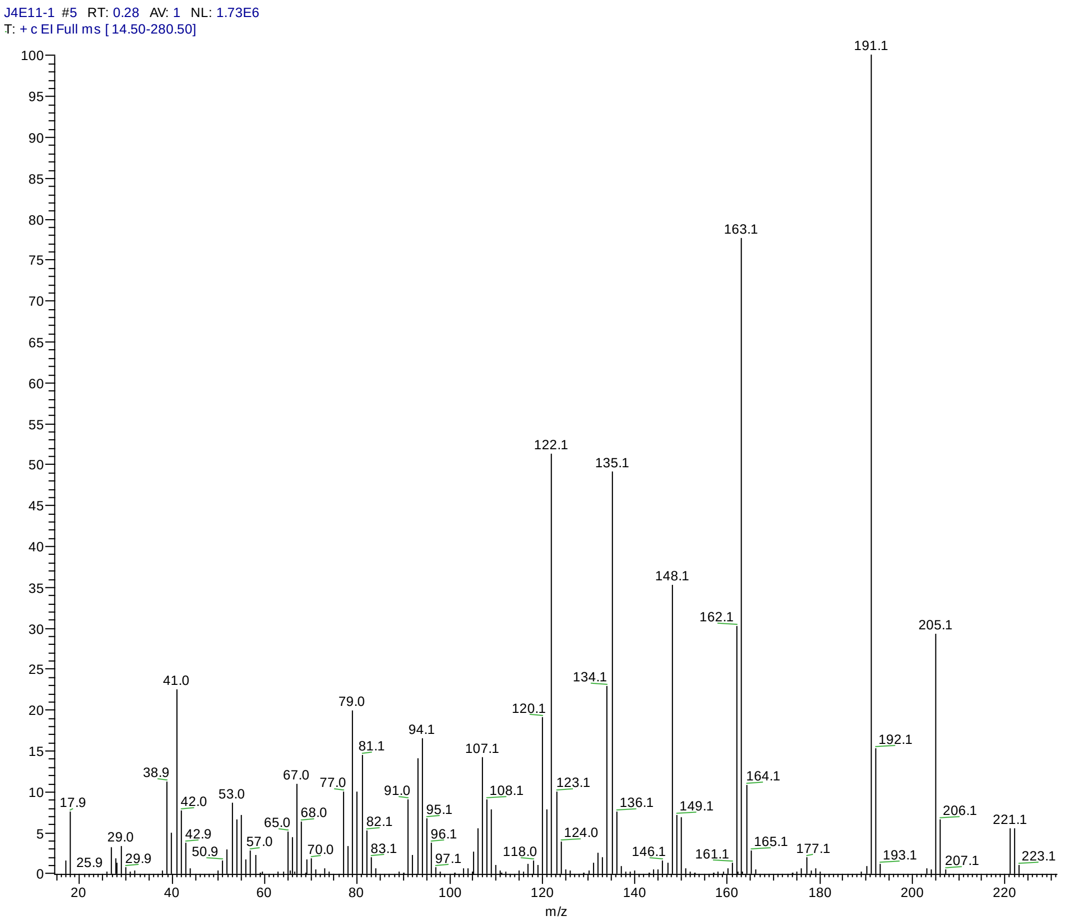

m/z	Intensity	Relative	m/z	Intensity	Relative
17.9	129821.0	7.49	109.1	135826.0	7.84
38.9	193542.0	11.17	120.1	331630.0	19.14
41.0	390672.0	22.55	121.1	135982.0	7.85
42.0	133369.0	7.70	123.1	171820.0	9.92
53.0	148734.0	8.59	134.1	395819.0	22.85

54.0	113286.0	6.54
55.0	123152.0	7.11
65.0	87821.0	5.07
67.0	188589.0	10.89
68.0	110322.0	30.78
68.0	109204.0	6.37
77.0	174121.0	10.05
79.0	345279.0	19.93
81.1	250477.0	14.46
91.0	156183.0	9.02
93.1	243185.0	14.04
94.1	285419.0	16.48
95.1	115977.0	6.70
96.0	244823.0	8.22
107.1	978645.0	14.13
108.1	155676.0	8.99
135.1	851539.0	49.16
136.1	130952.0	7.56
148.1	611590.0	35.31
149.1	124322.0	7.18
150.1	119135.0	6.88
162.1	524743.0	30.29
163.1	1345904.0	77.70
164.1	187419.0	10.82
191.1	1732286.0	100.00
192.1	264243.0	15.25
205.1	507975.0	29.32
206.1	114300.0	6.60
221.1	94957.0	5.48
222.1	94255.0	5.44
223.1	16612.0	0.96

Calculation of elemental compositions

Calculated value m/z= 221.1285 $(C_{12}H_{17}O_2N_2)^{+\cdot}$

Measured value m/z= 221.1286

Selected ESR *hfc* constants for radical 6c

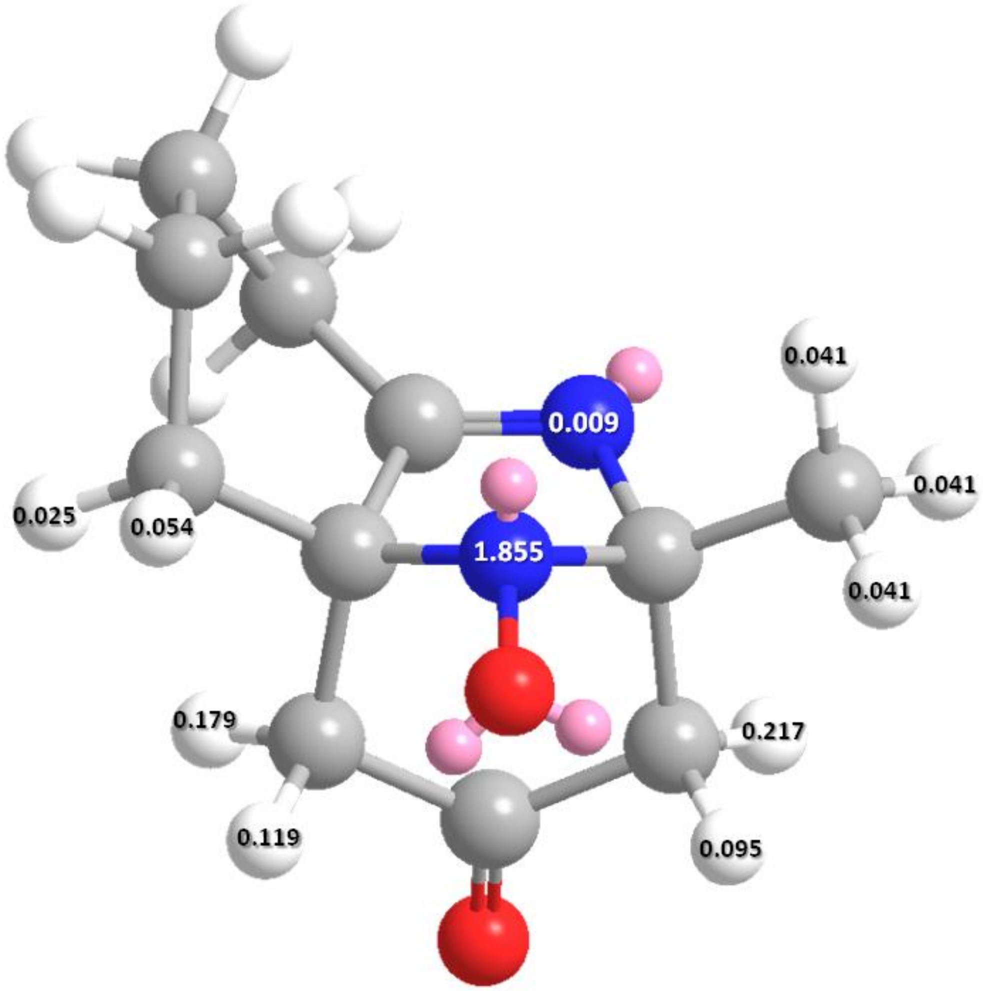

Fig. S14. Selected calculated constants of hyperfine interaction of an unpaired electron with hydrogen atoms and imine nitrogen in bicyclic nitroxide **6c**.
